# 左主支气管根部延长法治疗累及隆突的中央型肺癌（附3例报告）

**DOI:** 10.3779/j.issn.1009-3419.2011.03.13

**Published:** 2011-03-20

**Authors:** 旸凯 李, 澄澄 徐, 威 孙, 霓 张, 向宁 付

**Affiliations:** 430030 武汉，华中科技大学同济医学院附属同济医院普胸外科 Departmentof General Thoracic Surgery, Tongji Hospital Affiliated to Tongji Medical College, Huazhong University of Science andTechnology, Wuhan430030, China

**Keywords:** 肺肿瘤, 隆突, 胸部手术, Lung neoplasms, Carina, Thoracic surgery

## Abstract

**背景与目的:**

累及隆突的右侧中央型肺癌手术治疗较为困难。本研究旨在探讨左主支气管根部延长法治疗此类患者的有效性和可行性。

**方法:**

整块切除侵犯气管下段右侧壁的3例右上肺癌后，连续缝合左主支气管近端和气管下端，将左主支气管根部延长约3 cm，再将右中间段支气管与气管下端和左主支气管近端吻合，重建气道。

**结果:**

所有患者术后顺利脱呼吸机，排痰良好，无吻合口瘘等并发症，随访至今无肿瘤复发或转移，生活质量良好。

**结论:**

左主支气管根部延长法有利于右中间支气管与气管下端、左主支气管近端吻合重建，有利于术后排痰及降低吻合口瘘等并发症，适用于累及气管下段右侧壁，但左主支气管根部及右中间段支气管正常的右上叶中心肺癌。

## Abstract (en)

**Background and objective:**

Patient whose carina is involved by carcinoma is difficult to treat by surgery. The aim of this study is to evaluate the safety and effectiveness of left main bronchus root prolongation to cure these patients.

**Methods:**

Three patients with lung carcinoma received tumor, right upper lung and carina excision. And then the trachea and the carina was rebuilt by continuous suture, so that the left main bronchus root was extended by 3 cm, then the middle and lower lobe bronchus were sutured to the right lateral wall of the moved up eminence.

**Results:**

All the patients left hospital successfully after three-week treatment, without anastomotic stoma fistula. And they got good quality of life after 30, 21 and 11 months' follow-up, no recurrence or metabasis was found.

**Conclusion:**

The left main bronchus root prolongation can preserve the left lateral wall, however, part of the tracheal mucous membrane and arteria trachealis can be protected without injury. It's benifit for making productive cough and lowering complications after operation. The new carinal reconstruction process has definite indication, which refer to patient with normal left main bronchus root and the right inferior segment trachea involved by carcinoma.

中晚期肺癌占肺癌总数的一半以上，对于此类患者，外科治疗结合放化疗是目前较为有效的治疗方法。对于有手术指征的局部晚期非小细胞肺癌（non-small cell lung cancer, NSCLC），彻底切除肿瘤是提高长期生存率的关键。但是，部分患者由于肿瘤累及隆突或主支气管，或肿瘤虽局限但原发于气管下段、主支气管，需行气管隆突切除重建，此类手术创伤大、术后并发症高。最大限度地切除肿瘤组织的同时最大限度保留健康的肺组织，在保证根治性切除的前提下尽量缩小手术范围是肺外科的原则^[1]^。因此，改进手术方法，使其对正常组织损伤更小、术后并发症更少，对推动外科治疗NSCLC有重要意义。2007年12月以来华中科技大学同济医学院附属同济医院普胸外科对3例累及气管下段及右主支气管根部的右上肺癌患者，通过右上肺袖式切除加左主支气管根部延长法，对隆突实行部分切除并重建，获得成功，手术效果及长期随访均良好。现将治疗经验报告如下。

## 1 临床资料

### 1.1 一般资料

病例一：60岁男性患者，因“体检发现右肺肿块2周”入院。发病期间偶有咳嗽、咳痰，无进食梗阻。CT提示为右上肺纵隔旁软组织块影，考虑为纵隔型肺癌，纵隔内淋巴结肿大，最大直径为2 cm。术前检查未见转移病灶及手术禁忌症。术前诊断：右上肺纵隔型肺癌，累及气管、食管可能，临床分期CT4N2M0。

病例二：32岁男性患者，因“痰中带血1周”入院。CT提示为右主支气管根部新生物累及气管下段右侧壁。术前检查未见转移病灶及手术禁忌症。术前诊断：右主支气管肿瘤累及气管下段，临床分期CT4N0M0。

病例三：60岁男性患者，因“胸闷，心悸一月余”入院。纤支镜见右主支气管开口处新生物，累及气管下段。术前诊断：右主支气管肿瘤累及气管下段，临床分期CT4N0M0。

3例患者术前胸部CT见图 1。

**1:**
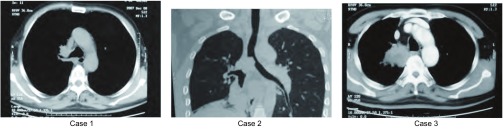
术前胸部CT示肿瘤累及隆突 The preoperative chest CT shows that carina is involved by lung carcinoma

### 1.2 手术方法

后外侧切口经第5、6肋间进胸，沿正常结构游离肿瘤组织，若发现食管肌层或部分上腔静脉壁受累应一并整块切除后重建（必要时可使用血管闭合器）。松解肺门，结扎右上肺各支血管并切断，清扫肺门、隆突下、上纵隔淋巴结。游离中间段支气管，在其起始部斜行横断。将累及隆突和气管下段的肿瘤组织连同右上肺整块移除，达到肉眼R0。由左主支气管起始处开始，向气管方向以4-0 Prolene缝线连续缝合隆突右侧壁及气管下段——相当于将左主支气管根部向气管方向延长；并根据气管下段缺损部分的大小，决定左主支气管延长的长度——直至缺损部分缩小到适合与中间段支气管吻合为止，一般需延长3 cm左右。然后将游离好的中间段支气管与剩余的气管缺损部分行端-侧吻合，吻合方法采用3-0 Prolene线连续缝合，需注意气管膜部相互对合防止扭转，缝完后线均匀收紧，线结打在气管壁外。以此方法重建后的隆突位置相当于向气管方向上移3 cm。气管吻合完毕，试水无漏气后，正中开腹，游离带蒂大网膜，经膈肌上提至胸腔内包埋吻合口，重建吻合口周围血供。最后留置胸管，逐层关腹及关胸。手术图片见图 2，手术示意图见图 3。

**2:**
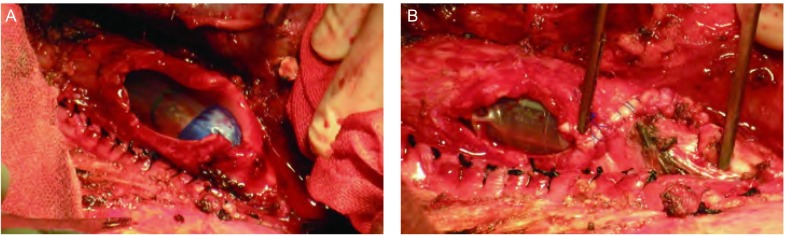
支气管根部延长术中图片。A：肿瘤切除后，可见气管内插管；B：左主支气管根部连续缝合延长3 cm后。 Pictures in left main bronchus root prolongation. A: Carcinoma is resected, and the endotracheal tube is showed; B: The left main bronchus root is prolonged 3 cm by continuous suture.

**3:**
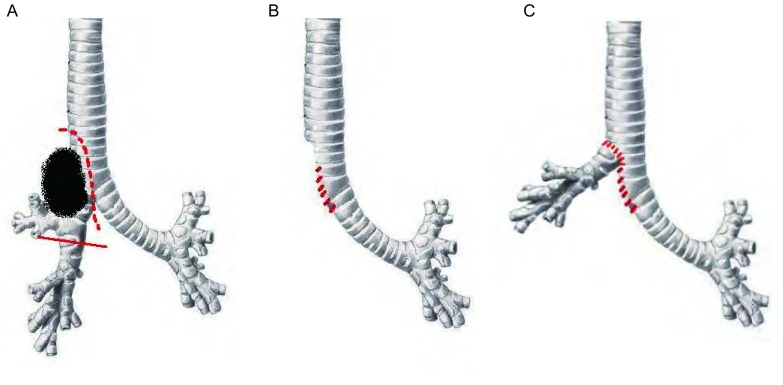
支气管根部延长术示意图。A：切除范围；B：左主支气管根部延长；C：中间段支气管端－侧吻合。 Delineation of left main bronchus root prolongation. A: the range of resection; B: the prolongation of left main bronchus root; C: the end-to-side anastomosis of the right intermediate bronchus to the left main bronchus.

## 2 结果

### 2.1 手术效果

病例一术毕给予气管插管，呼吸机辅助呼吸，4 h后顺利脱机。病例二、三手术结束即脱呼吸机返回病房。术后3例患者均多次床边纤支镜吸痰，可见吻合口愈合良好，无吻合口瘘。术后复查胸片示双肺扩张良好、无明显胸腔积液，1周后拔除胸管。术后2周行胸部CT三维重建见气管愈合良好、无狭窄（图 4）。术后3周顺利出院。术后1个月复查胸部CT，未见明显异常。

**4:**
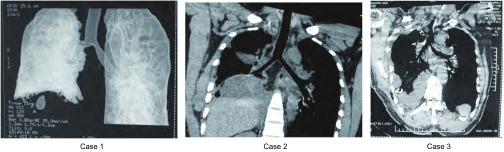
术后2周胸部CT三维重建 The chest CT and bronchial three-dimensional reconstruction on the 14^th^ postoperative day

### 2.2 病理类型及预后

病例一为右上肺高分化腺癌，气管壁和食管肌层受累，分期为PT4N2M0 R0。术后采用GC方案化疗4个周期，Pemetrexed方案化疗2个周期，及纵隔、肿瘤区放疗，随访30个月。病例二为右主支气管肉瘤样癌合并高分化鳞状细胞癌，癌巢直接蔓延累及主支气管旁淋巴结1枚及主支气管残端，分期为PT4N2M0 R1。术后采用GP方案化疗6个周期，及纵隔、肿瘤区放疗，随访21个月。病例三为右上肺高-中分化鳞状细胞癌，分期为PT4N0M0 R0，随访11个月。

3例患者术后随访至今均未见复发及转移，且生活质量良好。

## 3 讨论

传统的隆突成形术方法为分别切断气管下段和左、右主支气管，两主支气管先行侧-侧吻合，重建隆突，再与气管行对端吻合。由于隆突成形手术难度风险大、术后并发症和死亡率高及远期疗效难以肯定等因素^[2-5]^，隆突成形手术一直是胸外科医师所面临的挑战性问题。

术后吻合口瘘、吻合口狭窄是隆突成形术的主要并发症。主要与吻合口血供情况密切相关：因广泛游离和根治性的淋巴结清扫致使气管支气管吻合口血供不足、组织缺血坏死可致术后早期吻合口瘘，组织长期在慢性缺血状态下修复、过度增生，以及缺血造成的气管支气管软骨环破坏可导致吻合口狭窄^[4]^。因此在气管重建手术中，选择损伤最小的术式、重建被破坏的吻合口血供，是防止术后气道并发症的重要措施。

### 3.1 手术适应症的选择

左主支气管根部延长法适用于肿瘤侵犯右主支气管并累及气管下段的病例，但由于需要切除部分气管下段的侧壁并且重新吻合，故气管下段受累范围不能超过其周径的1/3，否则气管根部延长后将导致重建的“左主支气管”管口狭窄，远期吻合口瘢痕形成后，狭窄情况将进一步加重。因此对气管下段受累范围超过周径1/3的病例采用此种方法重建隆突需慎重。

采用支气管延长法需要严格把握手术适应症，对于术前CT见明显纵隔淋巴结肿大者，手术应慎重。Farjah等^[6]^回顾研究了1, 177例手术治疗T4期NSCLC的患者后认为，有无纵隔淋巴结转移是判断预后的独立因素，对IIIb期NSCLC、可切除的T4期患者预后优于N2/N3患者。3例患者虽分期较晚，但术前CT均未见明显肿大的纵隔淋巴结（病例一CT纵隔内仅见1枚2 cm大小淋巴结，该淋巴结病理镜下未见癌组织），术后随访均长期存活。因此我们认为，对于没有明显N2/N3淋巴结转移的T4期NSCLC患者，只要条件允许，采取积极有效的手术仍是首选治疗方法。

据现有经验表明，采用支气管延长法应尽量在气管间瘠肉眼下尚未受累的前提下进行。根据气管肿瘤手术范围的要求，通常认为气管支气管的切缘距瘤组织应 > 1 cm^[5]^，若气管间瘠受累，需要切除较多的隆突下壁，可能导致吻合后气管狭窄。由于隆突部分的气管管径较粗，故需要在实际情况下，根据术中观察决定是否采用左主支气管延长术。

### 3.2 术中需要注意的事项

因需要切除部分气管，故手术中一定强调使用双腔气管插管，并保证对位准确可靠。良好的麻醉配合可以保证在术中无需使用额外的远端支气管插管给氧。术中采用较低的潮气量、高频通气可以提供足够的血氧饱和度。在术中需要严密监测血气指标，及早纠正低氧血症和酸中毒。

气管吻合我们采用Prolene线连续缝合，缝合后线均匀收紧，避免切割，线结打在气管外。此方法手术时间短，操作简单。根据华中科技大学同济医学院附属同济医院普胸外科长期以来气管成形手术的实践证明，Prolene线单纯连续缝合吻合气管安全可靠，且不会增加手术并发症，但对术者的操作要求很高。

对于隆突成形的患者，多数学者认为以周围组织包绕吻合口，可有助于重建气管、支气管侧支血供，以减少因吻合口缺血造成的术后气道并发症。可选择的自体材料包括心包、胸膜、主动脉外膜、带蒂肌瓣、带蒂大网膜等，其中带蒂大网膜以血供丰富、组织填充性好、容易消除死腔等优点为首选，但右侧进胸需正中开腹游离大网膜和切开部分膈肌，增加了手术创伤。故是否使用带蒂大网膜需术者视术中情况决定，同时也需要更多临床病例的进一步观察总结。

### 3.3 支气管延长术的优点

与传统隆突成形方法相比，支气管根部延长术有如下的优点：①气管、支气管周围组织无需游离过多，避免了气管横断和再吻合，从而减少了对气管的损伤，最大限度地保留了气管粘膜下血管网的完整性，有效改善了吻合口周围、尤其是健侧支气管周围的血供；②与传统隆突成形方法相比，减少了一个吻合面（气管-左侧支气管），减少了吻合口并发症的发生几率，同时保护了气管左侧壁纤毛运动的连续性，有利于术后气道排痰；③左主支气管根部延长后，将隆突上移，可避免气管下段侧壁切除后直接缝合引起的气道狭窄；④右中间段支气管与气管下段侧壁吻合，重建的“隆突”上移后有利于右中下肺扩张、消除右上残腔。

总之，左主支气管根部延长法保留了隆突左侧壁的粘膜及血运，只是将“解剖性的隆突”上移，与传统全隆突重建术相比，有利于术后排痰及减少吻合口瘘等并发症，具有一定的先进性。其适用于累及气管下段右侧壁，但左主支气管根部及右中间段支气管正常的右上肺癌患者，有其特定的适应症。
